# Intentional sadness contagion: Social bonding with neural decoupling

**DOI:** 10.3758/s13415-026-01444-y

**Published:** 2026-05-19

**Authors:** Yanqiu Wei, Jinglu Chen, Siyuan Zhou, Ying Chen, Chao Kong, Zihao Zhang, Ping Hu

**Affiliations:** 1 Air Force Medical Center, Air Force Medical University, Beijing, China; 2 Department of Psychology, Renmin University of China, Beijing, China; 3 Turku PET Centre, Turku University Hospital and University of Turku, Turku, Finland; 4 Institute of Brain and Psychological Sciences, Sichuan Normal University, Chengdu, China; 5 School of Art Education, Hubei Institute of Fine Arts, Wuhan, China

**Keywords:** Intentional emotional contagion, Story sharing, Social interaction, Interpersonal neural synchronization, Emotions as Social Information Model

## Abstract

Intentional emotional contagion, the conscious transmission of emotions between individuals, has unclear neurobehavioral mechanisms in naturalistic dyadic communications. Experiment 1 demonstrated that conscious engagement enhanced sad emotional contagion between strangers. Using the fNIRS pseudo-hyperscanning technique, Experiment 2 showed a behavioral-neural dissociation in intentional sadness contagion: Behavioral findings revealed increased interpersonal intimacy and listener altruism. Neurocognitive findings demonstrated reduced activation in the left frontopolar cortex, bilateral dorsolateral prefrontal cortex (dlPFC), and angular/supramarginal gyri. While weaker interpersonal neural synchronization emerged between the speaker’s left frontopolar/dlPFC and listener’s right dlPFC. Mediation analysis indicated that intentional emotional contagion mediated emotional communication and self-other overlap, extending Emotions as Social Information Model. Findings suggest that intentional sadness contagion based on dyadic story sharing has an adaptive neurocognitive reorganization which facilitates prosocial behavior.

**Supplementary Information:**

The online version contains supplementary material available at 10.3758/s13415-026-01444-y.

## Introduction

Emotions possess inherent social properties and constitute an integral part of human social activity. They frequently emerge during interpersonal communication, are regulated by social norms and goals, and exert an influence on others (Keltner & Haidt, 1999; Parkinson et al., 2005; van Kleef, 2009). For example, sadness is correlated with internal feelings and psychological pain (Adolfi et al., 2017). The communication of sad emotions usually occurs among individuals in close relationships (Zaid et al., 2021). The experience of sadness can have beneficial effects on society, such as establishing the sense of group belonging (Porat et al., 2016).

Emotional contagion includes primitive emotional contagion and intentional emotional contagion. Primitive emotional contagion refers to the phenomenon of emotional convergence that occurs automatically, synchronously, and unconsciously (Hatfield et al., 1993; Spoor & Kelly, 2004). While intentional emotional contagion refers to the phenomenon of emotional convergence resulting from conscious association (Barsade, 2002; van Kleef & Côté, 2022). Perspective-taking and social evaluation involved in intentional emotional contagion, suggesting that it has a strong social interaction property.

As we know, research on social cognitive neuroscience has gradually evolved from the “single-brain observation” to an interpersonal communication mode of “multi-brain interaction” (Hasson et al., 2012), which abandoning the traditional single-observer perspective and being more capable of eliciting participants’ initiative and engagement during stimulus processing (Pan et al., 2022; Redcay & Schilbach, 2019; Schilbach et al., 2013). Moreover, telling short stories that occur in an individual’s life is considered an important part of human’s daily life and an important way to share emotions with others (Komulainen et al., 2021). Typically, listeners immersed in a story may have a strong emotional response (Komulainen et al., 2021). Thus, sharing stories that occur in one’s personal life (Smirnov et al., 2019) is a way to facilitate intentional emotional contagion.

To date, previous studies on intentional emotional contagion in social interactions have mostly been based on facial expression communication in the visual channel (Anders et al., 2011; Kinoshita et al., 2019), as well as story communication in the auditory channel (Smirnov et al., 2019). Moreover, previous studies have paid less attention to intentional emotional contagion among strangers (Anders et al., 2011; Kinoshita et al., 2019). However, interacting with strangers is a common and important part of human life, and acquaintance relationships mostly develop from stranger relationships. Therefore, this study explores strangers’ intentional emotional contagion through story sharing, which is based on the integration of multisensory channels.

In addition, with EEG and fMRI technique, it is impossible for pronounced affective behaviors, including facial expressions and body movements, to occur during interactions (Anders et al., 2011; Kinoshita et al., 2019; Smirnov et al., 2019). However, when intentional emotional contagion occurs during story sharing, it is naturally accompanied by facial expressions and body movements. At the same time, these nonverbal information plays an important role in social communication. In comparison, fNIRS technique has a high tolerance for motion artifacts (Wang et al., 2018). Moreover, the fNIRS hyperscanning technique has been widely applied in various social interaction scenarios such as interpersonal cooperation, conflict, creativity, learning, and emotional regulation (He et al., 2023; Jiang et al., 2012, 2015; Li et al., 2021, 2023; Liu et al., 2023; Long et al., 2021, 2022; Lu & Hao, 2024; Pan et al., 2022; Wang & Xu, 2025; Zhang et al., 2021, 2023). Therefore, the fNIRS hyperscanning technique is more suitable for exploring the neural mechanisms underlying intentional emotional contagion in social interactions.

To summarize, the present study used two experiments to investigate sad emotional contagion in social interactions. Experiment 1 explored the effect of conscious engagement on sad emotional contagion among strangers, to prove the existence of intentional emotional contagion. Based on Experiment 1, Experiment 2 used the fNIRS pseudo-hyperscanning technique to explore the neural mechanisms of intentional sadness contagion among strangers. At the same time, combined with the concept of “emotional expression affects observers’ behavior through emotional responses” in the emotion-as-social-information model (van Kleef & Côté, 2022; van Kleef, 2009, 2016), Experiment 2 also explored intentional emotional contagion on the interpersonal relationship between the interacting individuals.

## Experiment 1-the Effect of conscious engagement on emotional contagion between strangers

### Methods

#### Participants

In this experiment, we used the “sender-receiver” mode. As we know, female is more accurate than male in recognizing subtle facial expressions (Hoffmann et al., 2010). Therefore, all the listeners in this experiment were females.

After the pilot study, we selected two speakers (24.00 ± 1.41 years (*M* ± *SD*)) as the formal speakers. A total of 41 listeners were recruited for this experiment and were divided into two groups: direct-viewing and perspective-taking. One participant who failed to understand the instructions correctly was deleted, resulting in 20 valid listeners (20.05 ± 2.14 years (*M* ± *SD*)) in direct-viewing group and 20 valid listeners (20.60 ± 2.28 years (*M* ± *SD*)) in perspective-taking group.

All participants had normal or corrected-to-normal vision, and no history of brain injury or mental disorders. The participants signed an informed consent form before the experiment and received course credit or monetary compensation after the experiment. This study was approved by the Ethics Committee (IRB approval number: 23–006, 23–039) of the Department of Psychology, Renmin University, China.

#### Experimental design

This study used a two-factor mixed experimental design. The within-subject independent variable was emotion type, categorized into neutral and sad emotions. The between-subjects independent variable was the level of conscious engagement, categorized into direct-viewing and perspective-taking groups. The dependent variable was the listeners’ self-assessment of their emotional state.

#### Pilot study: Screening videos of neutral and sad stories

**Fig. 1:**
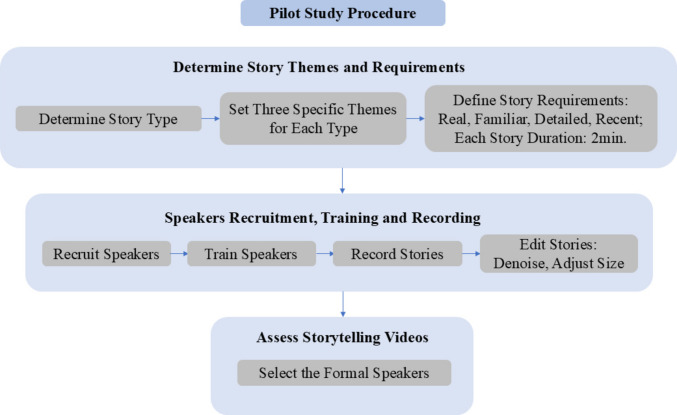
Pilot study procedure

The experimental paradigm of this study was based on the “sender-receiver” communication mode (Wei et al., 2024). One tells a real story that happened in her own life, while the other listens to the story and can make nonverbal responses. Videos of stories must be prepared and screened in advance (Jospe et al., 2020). All speakers received money compensation after the pilot study. The pilot study procedure included the determination of the story’s themes and requirements, speakers’ recruitment, training, and recording, as well as the assessment of storytelling videos (Fig. 1).

Specifically, the themes of the neutral stories are “My Routine Day; Cleaning; Self-study.” For the sad stories, the themes are set as “Relatives’ illness or death; Alienation or breakdown of friendship; Failure in examinations.” Each theme is associated with a distinct short story. The narrative duration of each story should be two minutes (El Haj et al., 2021). The story must be familiar to college students (Gandolphe et al., 2018) and should be real-life events experienced by themselves. The description of each story should be detailed, including the time and place of occurrence and the emotional experiences of the speaker and people involved (El Haj et al., 2021). Moreover, these events should have occurred recently (D’Argembeau & van der Linden, 2004). The duration of each story was two minutes (El Haj et al., 2021).

Speakers were recruited and trained. They should be skilled at conveying their emotions. The training protocol included: initial feedback during face-to-face interviews, submission of revised audio recordings, refinement feedback for each story. The feedback focused on speaker’s delivery skills, such as vividness of story’s details, facial expressions, vocal tones, and body language. During the recording phase, participants narrated these stories in their own words. We then used the Adobe Premiere 2020 software to briefly edit the videos. This editing aimed to remove the background noise and adjust it to an appropriate size.

Subsequently, seventeen female participants (one voluntarily withdrew from the experiment, resulting in sixteen valid evaluators, 20.44 ± 2.48 years (*M* ± *SD*)) were recruited to evaluate speakers’ video materials. The evaluation criteria consisted of five items: Q1: Evaluate the speaker’s physical attractiveness; Q2: Evaluate the speaker’s naturalness of storytelling; Q3: Evaluate the degree of emotional identification from the perspectives of both speaker and listener; Q4: Evaluate the speaker’s own emotional valence; and Q5: Evaluate the listener’s emotional valence induced by the story. Consequently, two female speakers (24.00 ± 1.41 years (*M* ± *SD*)) were selected as the formal speakers. Appendix 1 presents the specific results.

#### Experimental procedure

**Fig. 2:**
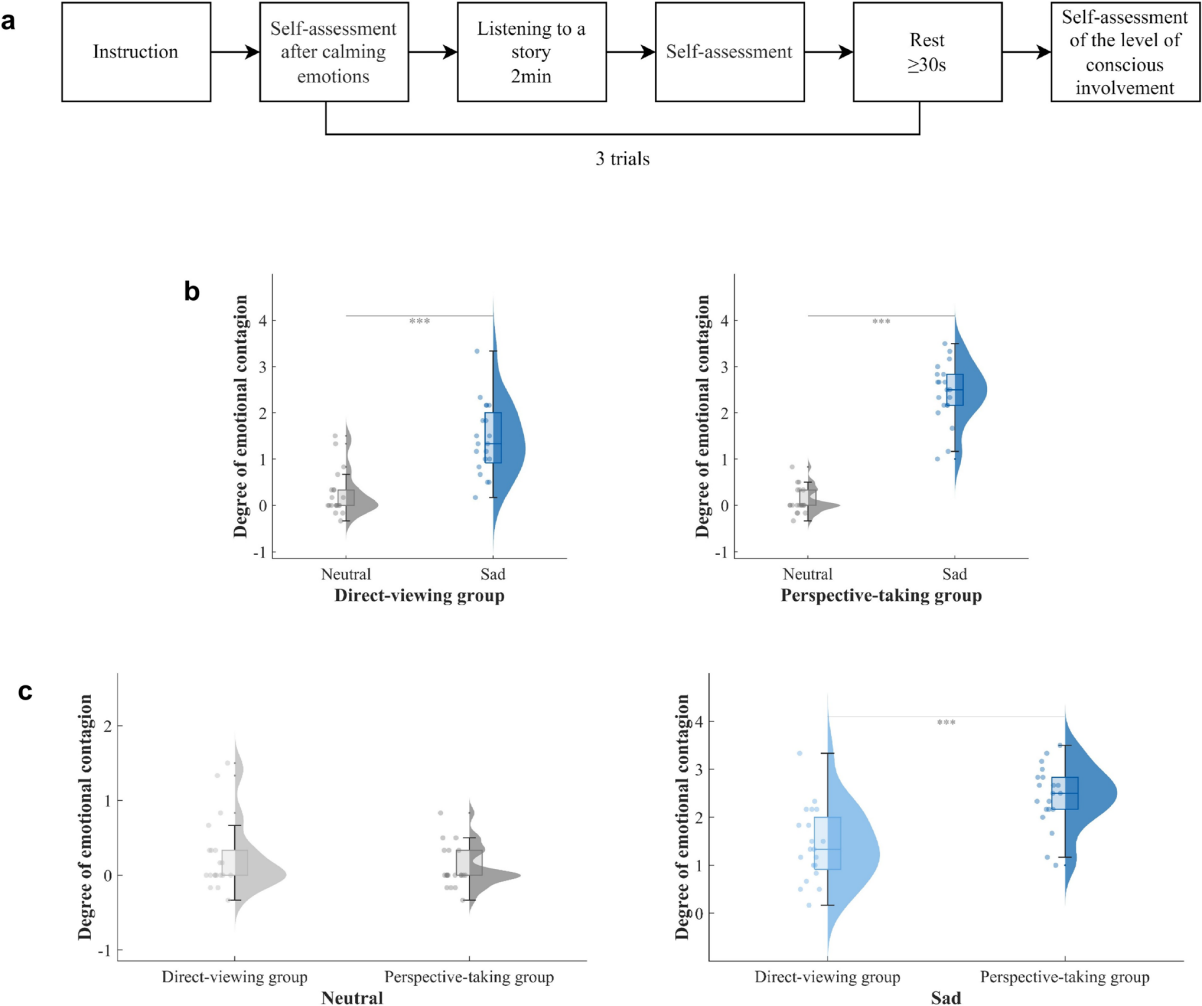
Procedure and behavioral results of Experiment 1. (**a**) The procedure in Experiment 1; (**b**) Degree of emotional contagion induced by stories in Experiment 1; (**c**) Degree of emotional contagion induced by groups in Experiment 1. Note: ***p < 0.001

In this phase, videos were presented to the listeners, and the behavioral data of listeners were collected simultaneously. The experimental procedure for the listeners (Fig. 2a) was similar to the speakers’ recording procedure. Before listening to the stories, listeners were required to adjust their emotions to a neutral state and then evaluate their current emotional experiences on a nine-point Likert scale (1 = extremely unpleasant, 5 = a neutral emotion, and 9 = extremely pleasant). Subsequently, a preparation cue appeared at the screen’s center. Each video lasted for 2 min. After watching each video, listeners evaluated their emotional experiences on a nine-point Likert scale (Peng et al., 2021).

The participants were divided into two groups. Sad and neutral stories correspond to one block. Within each block, the order of stories was randomized. The total duration of the experiment was approximately 40 min. When watching the videos, the instructions for the two groups (Galinsky & Ku, 2004; Zhong et al., 2015) were as follows:

Direct-viewing group: Please listen directly to the story told by the speaker and complete an evaluation task that follows the story. Do not think about the evaluation task during the listening stage.

Perspective-taking group: Please put yourself in the speaker’s position and experience her emotions and then complete an evaluation task after listening to the story. Do not think about the evaluation task during the listening stage.

### Results

Bonferroni correction was used for all post-hoc comparisons (Andrade, 2019; VanderWeele & Mathur, 2019) involved in this experiment.

#### Manipulation of the degree of conscious engagement

To examine the validity of the degree of conscious engagement, one-way ANOVA analyses were conducted on listeners’ self-assessment scores for perspective-taking. The results suggested that the perspective-taking manipulation was valid (*F*(1, 38) = 61.11, *p* < 0.001, η^2^_p_ = 0.62; Direct-viewing group: 4.59 ± 1.64, Perspective-taking group: 7.64 ± 0.59, *M* ± *SD*).

#### Degree of emotional contagion induced by emotion type

To compare the degree of emotional contagion induced by the different stories, a two-way repeated-measures ANOVA was conducted. The within-subject independent variable was emotion type (neutral or sad emotion), and the between-subject independent variable was the level of conscious engagement (direct-viewing group or perspective-taking group). The dependent variable was the change in listeners’ self-assessment of their emotional state. For neutral stories, the score was calculated as the self-assessment score after listening minus the self-assessment score before listening. For sad stories, the score was calculated as the self-assessment score before listening minus the self-assessment score after listening. The results showed that the main effect of emotion type was significant (*F*(1, 38) = 227.86, *p* < 0.001, η^2^_p_ = 0.86). The interaction between emotion type and level of conscious engagement was also significant (*F*(1, 38) = 24.84, *p* < 0.01, η^2^_p_ = 0.4). The results of the simple effect indicated that in the direct-viewing group, there was a significant difference between listening to neutral stories and sad stories (*p* < 0.001). In the perspective-taking group, there was also a significant difference between listening to neutral stories and sad stories (*p* < 0.001) (Fig. 2b). When listening to neutral stories, there was no significant difference between the two groups (*p* > 0.05); however, when listening to sad stories, there was a significant difference between the two groups (*p* < 0.001) (Fig. 2c). The results of the descriptive statistics are shown in Table 1.

**Table 1:** Variation in the degree of listener’s emotional contagion of Experiment 1

Emotion type	Level of conscious engagement (*M* ± *SD*)	
	Direct-viewing group	Perspective-taking group
Neutral	0.25 ± 0.49	0.10 ± 0.28
Sad	1.43 ± 0.77	2.43 ± 0.65

## Experiment 2-Intentional emotional contagion between strangers: A fNIRS pseudo-hyperscanning technique

### Methods

#### Participants

**Fig. 3:**
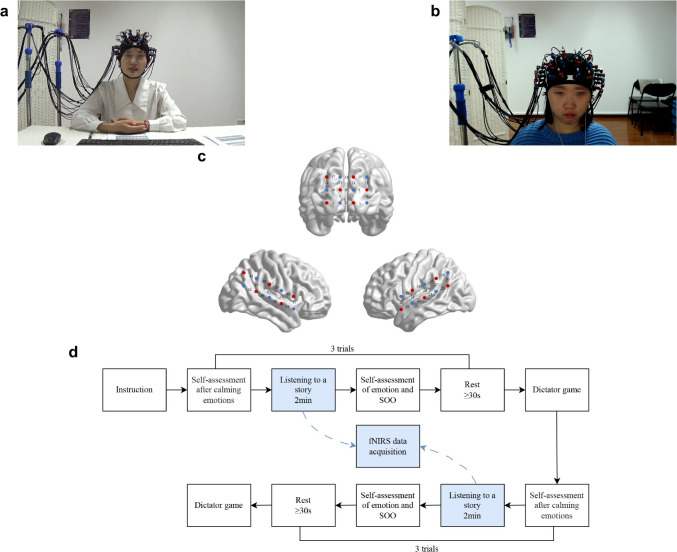
Experimental setup, probe location and procedure of Experiment 2. (**a**) Setup for speaker data acquisition in Experiment 2; (**b**) Setup for listener data acquisition in Experiment 2; (**c**) The optode probe set in Experiment 2 (placed on the bilateral frontal, temporal, and parietal cortices); (**d**) The procedure of each block in Experiment 2

In this experiment, we also used the “sender-receiver” mode. Similar to Experiment 1, all the listeners in this experiment were female. The experimental materials used were the same as those used in Experiment 1. Brain signals were recorded during the storytelling (Fig. 3a). Thirty-four listeners were recruited for Experiment 2, one of whom was interrupted because the participant felt unwell. Finally, brain and behavioral data from 33 valid listeners (20.67 ± 2.46 years (*M* ± *SD*)) were retained (Fig. 3b).

All participants had normal or corrected-to-normal vision, and no history of brain injury or mental disorders. The participants signed an informed consent form before participating in the experiment and received monetary compensation after the experiment. This study was approved by the Ethics Committee (IRB approval number: 23–006, 23–039) of the Department of Psychology, Renmin University, China.

#### Experimental design

In this study, the within-subject independent variable was emotion type, categorized into neutral and sad emotions. The dependent variables can be categorized into three aspects: behavioral indicators, which refer to the self-evaluations of the listener’s own emotional state and the perceived relationship between themselves and speakers; single-brain indicators, which refer to the listeners’ activation level in each channel; and interpersonal neural synchronization (INS) indicators, which represent the INS values of two interacting individuals.

#### Experimental procedure

The brain activity of the listeners was continuously recorded using a LABNIRS system (Shimadzu Corporation, Kyoto, Japan) (Fig. 3c). The procedure for the listener was similar to that used for the speaker. Before listening to the story, listeners were required to adjust their emotions to a neutral state and then use a nine-point scale to assess their emotions. The preparation cues for story listening then appeared in the screen’s center. Each story video lasted for 2 min. While watching the videos, listeners were required to engage in feeling speakers’ emotions from the perspective of speakers to facilitate the occurrence of intentional emotional contagion.

After watching each video, listeners evaluated their emotional experience on a nine-point Likert scale (1 represented extremely unpleasant, 5 represented a neutral emotion, and 9 represented extremely pleasant) and a six-point Likert scale to assess the degree of self-other overlap (SOO) between the speaker and listener. After conversion, the larger the value, the more intimate is the relationship between them (Aron et al., 1991, 1992; Peng et al., 2021).

Prior to story sharing, a 5-min resting-state data were collected. The story sharing phase was divided into two blocks: one for sad stories and the other for neutral stories. After three stories with the same emotion, the listeners were asked to complete the dictator’s game (Cameron et al., 2013).

The instructions for the dictator game are as follows:

- If you receive 200 RMB, give some to yourself and the rest to the current speaker.
- Please allocate the amount of money.

The blocks corresponding to the sad and neutral stories were counterbalanced between the listeners. For each listener, the sequences of the two speakers within each block were randomized. Moreover, the order of stories of the same emotion within each speaker was randomized. To some extent, this reduced the mutual contamination caused by the change of speakers and also avoided the sequential effect of the stories’ valence. The duration of the entire experiment was approximately 1.5 ~ 2 h. The specific procedure for each block is illustrated in Fig. 3d.

#### fNIRS data acquisition

As the participants viewed the videos, data were continuously recorded using the LABNIRS system. Two main nervous systems, the mirror neuron system and the mentalizing system (Kingsbury & Hong, 2020; Wang et al., 2018) may be involved in intentional emotional contagion in social interactions. Similar to a previous study (Wei et al., 2024), three sets of optode probes covered the frontal, temporal, and parietal cortices, with 16 laser sources and 16 laser detectors, corresponding to a 43-channel montage (Fig. 3c). The distance between the two optode probes was 3 cm, which took measurements approximately 15–25 mm beneath the scalp (Hoshi et al., 2005; Okada & Delpy, 2003). The distance between the listener and screen was approximately 70 cm.

Three wavelengths of 780, 805, and 830 nm were used to measure hemodynamic changes in blood oxyhemoglobin (HbO) and deoxyhemoglobin (HbR). The signals were recorded at a sampling rate of 9.5238 Hz. Prior to the experiment, the signal quality was adjusted and calibrated using the LABNIRS system to obtain stable and high-quality data signals. After the experiment, researchers used a 3D locator to obtain the positions of each probe. Finally, the average physical positions of all channels were obtained to determine the average physical positions of each channel in this experiment.

#### Single-brain activation statistics

In this experiment, functions in the MATLAB 2013b software were used to analyze the fNIRS data. The concentration changes of HbO, HbR, and HbT were evaluated based on the modified Beer-Lambert law. As HbO concentration changes are highly sensitive to cerebral blood changes and the signal-to-noise ratio (Hoshi, 2007), only the change in the HbO concentration was used in this experiment. For each listener, a General Linear Model (GLM) analysis was conducted for each channel. The hemodynamic response function filter and wavelet-minimum description length detrending algorithm were employed to eliminate physical noise and artifacts (Jang et al., 2009). For each condition of each listener, the β coefficient values of the GLM were extracted from different trials as weights to represent brain activity (Xie et al., 2018). In addition, we removed the outliers of single-brain activation. For example, the 14th listener reported not being fully engaged in the experiment and showed obviously pleasant expressions while listening to the first neutral story. Consequently, this trial was deleted. The statistics of the behavioral results also excluded this trial.

Subsequently, the behavioral results and single-brain activation results were analyzed using IBM SPSS Statistics 26. One-way repeated ANOVA analyses were conducted on the HbO β values of each listener under each condition and channel. Finally, we only reported *p*-values corrected by Bonferroni correction as significant results.

#### INS statistics

**Fig. 4:**
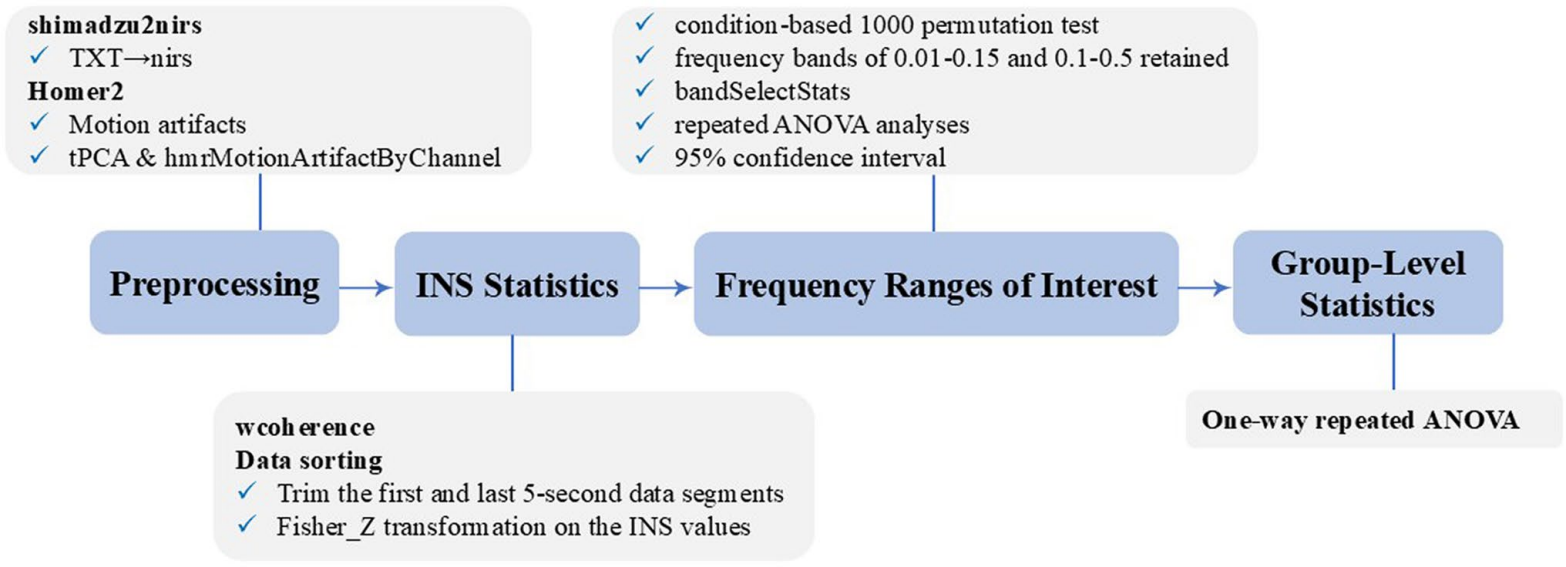
INS statistics flow of Experiment 2

Specific statistic steps (Fig. 4) are as follows:

##### Preprocessing

The MATLAB2020a software was used to analyze the fNIRS pseudo-hyperscanning data. First, using the shimadzu2nirs function, the Shimadzu TXT files were converted to nirs format files that Homer2 can read. Subsequently, 33 nirs format files were preprocessed. We referred to Li et al., (2021, 2023) to set the specific parameters. Motion artifacts were recognized and corrected using tPCA (function: hmrMotionCorrectPCArecurse; input parameters: tMotion = 0.5, tMask = 1, STDthresh = 30, AMPthresh = 0.5, nSV = 0.97, maxIter = 5). Possible artifacts were further reduced using hmrMotionArtifactByChannel (tMotion = 0.5, tMask = 1, STDEVthresh = 30, AMPthresh = 0.5) and hmrMotionCorrectSpline (*p* = 0.99).

##### INS statistics

To assess the INS between two paired data segments, the “wcoherence” function in MATLAB was used to calculate the wavelet transform coherence (WTC). Trim the first and last 5-s data segments form each trial. Subsequently, Fisher_Z transformation was performed on the INS values. Moreover, the INS value corresponding to the fourteenth listener was deleted for the same reason as in the single-brain activation statistics. The INS values obtained by all participants in each condition were averaged, resulting in 33 INS values corresponding to the neutral emotional condition and 33 INS values corresponding to the sad emotional condition.

##### Determining the frequency ranges of interest

Following the methods of Long et al., (2021, 2022), we used a condition-based 1,000 permutation test to identify the frequency bands of interest. During each time, 33 values were randomly selected to serve as the values corresponding to the null neutral emotion, and the remaining 33 INS values were considered as the values corresponding to the null sad emotion.

Subsequently, one-way repeated ANOVA analyses were conducted for each frequency point and channel. The frequency bands of 0.01–0.15 and 0.1–0.5 were retained (Baker et al., 2016; Liu et al., 2015; Long et al., 2021, 2022), and null values were assigned to the remaining frequency bands. Consequently, the *p*_map and *F*_map corresponding to this sampling was obtained. The bandSelectStats function was then employed to identify the longest frequency band and its corresponding channel pair. The INS value corresponding to the channel pair and frequency band was extracted. Finally, repeated ANOVA analyses corresponding to this null sample were performed. Consequently, a null *F*-value and null *p*-value were obtained from this null sampling. After 1,000 repetitions, 1,000 *F*-values were obtained.

The differences between the conditions for all continuous frequency bands and their corresponding channel pairs in real samples were analyzed. As a result, 3,893 *F*-values were obtained. Subsequently, we examined each *F*-value separately to determine whether it lay to the right of the 95% confidence interval. If the *F*-value fell on the right side of the 95% confidence interval, it would be retained. The longest frequency band was used as the final frequency band of interest.

##### Group-level statistics

One-way repeated ANOVA was conducted to analyze the differences between the conditions under the frequency band of interest and its corresponding channel pairs.

### Results

In this experiment, we examined the differences in the degree of intentional emotional contagion, self-other overlap, prosocial behaviors, single-brain activation, and INS, respectively, induced by emotion type, and constructed a mediation model. Bonferroni correction was used for all post-hoc comparisons (Andrade, 2019; VanderWeele & Mathur, 2019).

#### Degree of intentional emotional contagion induced by emotion type

**Fig. 5:**
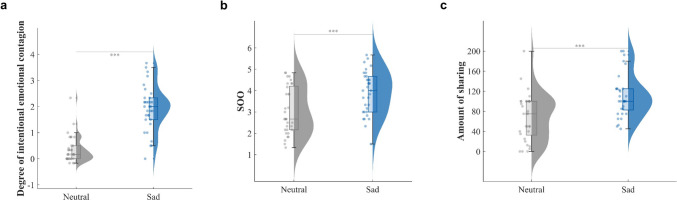
Behavioral results of Experiment 2. (**a**) Degree of intentional emotional contagion induced by stories in Experiment 2; (**b**) Self–other overlap induced by emotion types in Experiment 2; (**c**) Amount of sharing induced by emotion types in Experiment 2. Note: ***p < 0.001

To compare the degree of intentional emotional contagion induced by emotional stories, we conducted one-way repeated ANOVA. The independent variable was emotion type (neutral or sad emotions), and the dependent variable was the change in listener’s self-assessment of emotional state. For neutral stories, the score was calculated as the self-assessment score after listening minus the self-assessment score before listening. For sad stories, the score was calculated as the self-assessment score before listening minus the self-assessment score after listening. The results showed that the main effect of emotion type was significant (*F*(1, 32) = 90.07, *p* < 0.001, η^2^_p_ = 0.74). The degree of intentional emotional contagion induced by sad stories (*M* ± *SD*: 1.93 ± 0.86) was greater than that induced by neutral stories (*M* ± *SD*: 0.35 ± 0.51; Fig. 5a).

#### Self–other overlap induced by emotion types

To compare the differences in self-other overlap between the speaker and listener as perceived by the listener, a one-way repeated ANOVA was conducted. The independent variable was emotion type (neutral or sad emotion). The dependent variable was the listener’s self-assessment of self-other overlap after listening to each story. The results showed a significant main effect of emotion type (*F*(1, 32) = 16.51, *p* < 0.001, η^2^_p_ = 0.34). The degree of self-other overlap induced by sad stories was greater than neutral stories (*M* ± *SD*: 3.91 ± 1.01 for sad stories, 3.05 ± 1.1 for neutral stories; Fig. 5b).

#### Prosocial behaviors induced by emotion types

To compare differences in listeners’ prosocial behaviors after they listened to different stories, a one-way repeated ANOVA analysis was conducted. The independent variable was emotion type (neutral or sad emotions), and the dependent variable was the amount of money that the listener wanted to allocate after listening to three stories. The results are shown in Fig. 5c, and the main effect of emotion type was significant (*F*(1, 32) = 19.88, *p* < 0.001, η^2^_p_ = 0.38). After listening to three sad stories, the amount of money that the listeners allocated to the speakers was significantly greater than that after listening to neutral stories (*M* ± *SD*: 109.77 ± 41.9 for sad stories and 68.41 ± 46.92 for neutral stories, *p* < 0.001).

#### Single-brain activation induced by emotion type

**Fig. 6:**
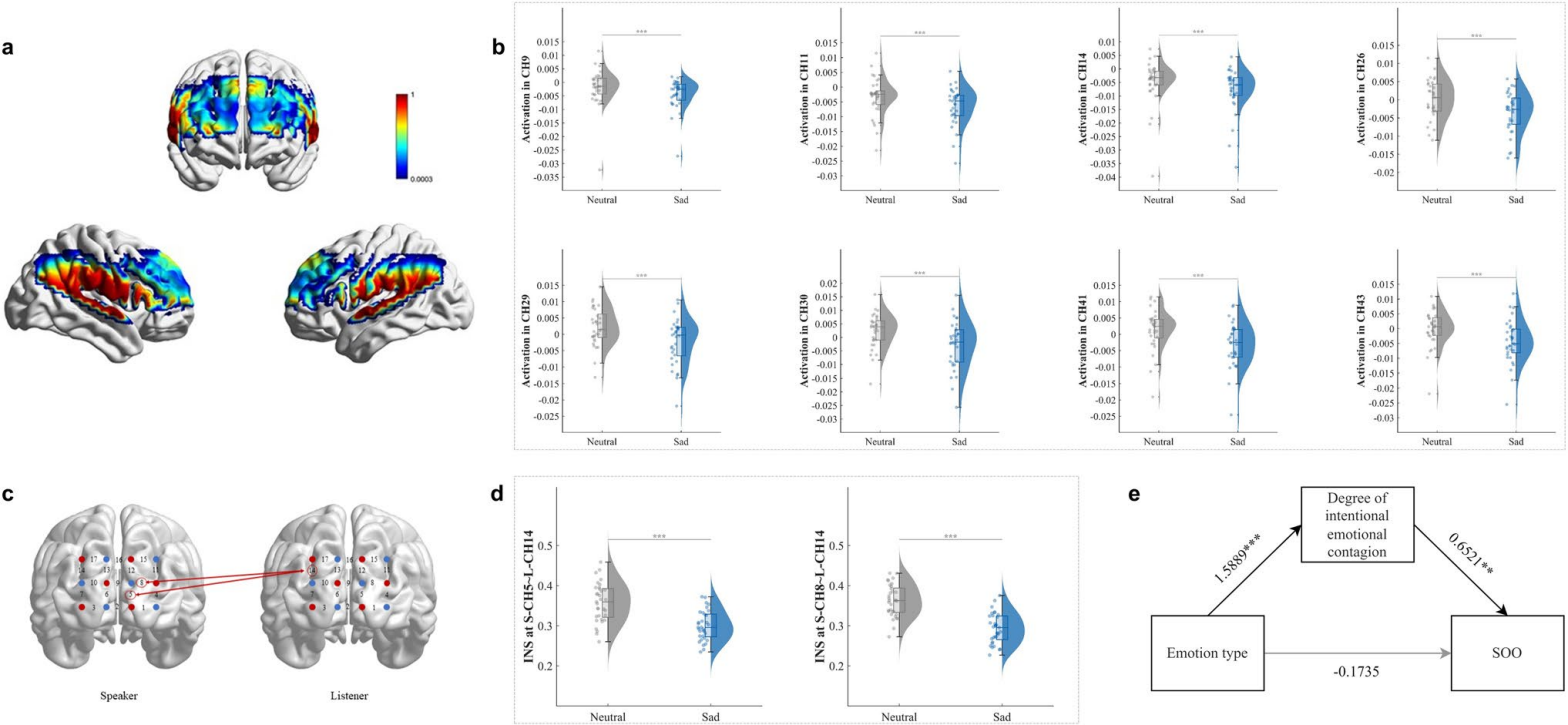
Single-brain activation, INS and behavioral mediation of Experiment 2. (**a**) Single-brain activation in Experiment 2; (**b**) Statistics results of single-brain activation induced by emotion types in Experiment 2; (**c**) Significant channel pair for INS in Experiment 2; (**d**) Statistics results of INS induced by emotion types in Experiment 2; (**e**) Emotion type and the degree of self-other overlap: the mediating function of the degree of intentional emotional contagion. Note: **p < 0.01; ***p < 0.001

To compare the differences in single-brain activation induced by emotion type, one-way repeated ANOVA was conducted. The independent variable was emotion type (neutral or sad emotion). The dependent variable was the β-coefficient value for each listener under each condition and in each channel. A significant main effect of emotion type was found in channel 9 (*F*(1, 32) = 18.1, *p* < 0.001, η^2^_p_ = 0.36), channel 11 (*F*(1, 32) = 14.75, *p* = 0.001, η^2^_p_ = 0.32), channel 14 (*F*(1, 32) = 20.66, *p* < 0.001, η^2^_p_ = 0.39), channel 26 (*F*(1, 32) = 23.13, *p* < 0.001, η^2^_p_ = 0.42), channel 29 (*F*(1, 32) = 20.31, *p* < 0.001, η^2^_p_ = 0.39), channel 30 (*F*(1, 32) = 16.71, *p* < 0.001, η^2^_p_ = 0.34), channel 41 (*F*(1, 32) = 28.74, *p* < 0.001, η^2^_p_ = 0.47), and channel 43 (*F*(1, 32) = 22.43, *p* < 0.001, η^2^_p_ = 0.41). Moreover, the activation patterns were similar across the channels (see Fig. 6a for single-brain activation and Table 2 for the corresponding brain regions). Specifically, neutral stories evoked significantly greater activation than sad stories (Channel 9: − 0.002 ± 0.007 for neutral stories and − 0.004 ± 0.006 for sad stories; Channel 11: − 0.003 ± 0.006 for neutral stories and − 0.006 ± 0.006 for sad stories; Channel 14: − 0.005 ± 0.008 for neutral stories and − 0.008 ± 0.008 for sad stories; Channel 26: 0.000 ± 0.005 for neutral stories and − 0.003 ± 0.006 for sad stories; Channel 29: 0.002 ± 0.006 for neutral stories and − 0.002 ± 0.007 for sad stories; Channel 30: 0.002 ± 0.006 for neutral stories and − 0.003 ± 0.009 for sad stories; Channel 41: 0.001 ± 0.006 for neutral stories and − 0.003 ± 0.007 for sad stories; Channel 43: − 0.000 ± 0.006 for neutral stories and − 0.004 ± 0.007 for sad stories *M* ± *SD*, Fig. 6b).

**Table 2:** The brain regions corresponding to CH9 CH11 CH14 CH26 CH29 CH30 CH41 CH43

Channel	AAL and coverage	Brodmann area and coverage
CH9	Frontal_Sup_Medial_L, 0.6692	10—Frontopolar area, 0.71103
CH11	Frontal_Mid_L, 0.80374	9—Dorsolateral prefrontal cortex, 0.54673; 46—Dorsolateral prefrontal cortex, 0.45327
CH14	Frontal_Mid_R, 0.82243	9—Dorsolateral prefrontal cortex, 0.65421
CH26	Parietal_Inf_L, 0.56401; SupraMarginal_L, 0.43599	40—Supramarginal gyrus part of Wernicke's area, 0.9827
CH29	Angular_L, 0.74583	39—Angular gyrus, part of Wernicke's area, 0.87917
CH30	Angular_L, 0.51948; Occipital_Mid_L, 0.4329	39—Angular gyrus, part of Wernicke's area, 0.62771
CH41	Angular_R, 0.90411	39—Angular gyrus, part of Wernicke's area, 0.73973
CH43	Angular_R, 0.61111	19—V3, 0.40598

#### INS induced by emotion type

In the real samples of Experiment 2, the *F*-values of 206 continuous frequency bands fell to the right of the 95% confidence interval of 1,000 random samples. The longest frequency band, from 0.023 to 0.1463 Hz, was selected as the frequency band of interest. Subsequently, a difference statistic of the INS values between conditions was conducted for the frequency band of interest and its corresponding channel pair (the channel pair consisting of speaker’s CH5 and listener’s CH14, as well as the channel pair consisting of speaker’s CH8 and listener’s CH14. The corresponding brain regions were listed in Table 3). We performed a one-way repeated ANOVA. The independent variable was emotion type (neutral or sad emotion), and the dependent variable was the INS value corresponding to each channel pair.

**Table 3:** Brain regions corresponding to INS of Experiment 2

Channel pair	AAL and coverage	Brodmann area and coverage
S_CH5 ~ L_CH14	S: Frontal_Sup_L, 0.69153; L: Frontal_Mid_R, 0.82243	S: 10-Frontopolar area, 1; L: 9-Dorsolateral prefrontal cortex, 0.65421
S_CH8 ~ L_CH14	S: Frontal_Sup_L, 0.752; L: Frontal_Mid_R, 0.82243	S: 46-Dorsolateral prefrontal cortex, 0.424; L: 9-Dorsolateral prefrontal cortex, 0.65421

The results indicated a significant main effect of emotion type on the S_CH5 ~ L_CH14 channel pair (Fig. 6c) (*F*(1, 32) = 43.39, *p* < 0.001, η^2^_p_ = 0.58). The INS values induced by neutral stories were significantly greater than those induced by sad stories (neutral stories: 0.358 ± 0.048; sad stories: 0.3 ± 0.037 (*M* ± *SD*). Similarly, for the S_CH8 ~ L_CH14 channel pair (Fig. 6c), a significant main effect of emotion type was also observed (*F*(1, 32) = 52.17, *p* < 0.001, η^2^_p_ = 0.62). The results suggested that the INS values elicited by neutral stories were significantly higher than those evoked by sad stories (neutral stories: 0.362 ± 0.04; sad stories: 0.293 ± 0.037 (*M* ± *SD*); Fig. 6d).

#### Emotion type and self–other overlap: The mediating role of intentional emotional contagion

Experiment 2 integrated the “Emotions as Social Information Model” (van Kleef & Côté, 2022; van Kleef, 2009, 2016) and the “Self − Other Overlap Theory” (Aron et al., 1991, 1992). As the interaction between the speakers and listeners deepened, the listener may feel an increasing sense of intimacy between them. This study investigated whether the degree of intentional emotional contagion experienced by listeners mediated the relationship between speakers’ emotional expression and the degree of self-other overlap perceived by listeners. Because emotion type is the independent variable, statistical analysis was conducted using the memore plugin in SPSS (Montoya & Hayes, 2017). A bias-corrected bootstrap test (with 5,000 resamples) was used. Detailed results are shown in Fig. 6e.

Emotion type significantly predicted the degree of intentional emotional contagion (path coefficient = 1.5889, *p* < 0.001), while emotion type did not significantly predict the degree of self-other overlap (path coefficient = − 0.1735, *p* > 0.05). The degree of intentional emotional contagion significantly predicted the degree of self-other overlap (path coefficient = 0.6521, *p* < 0.01). The path coefficient of the total effect size was 0.8626 (*p* < 0.001). Based on the bias-corrected bootstrap test, the 95% confidence interval of the mediating effect of the degree of intentional emotional contagion between emotion type and self-other overlap was [0.1007, 1.7308], indicating that listeners’ engagement in intentional emotional contagion mediated the relationship between speakers’ emotional expressions and self-reported self–other overlap.

### Discussion

Social interaction is an integral part of daily life (Dumas, 2011; Redcay & Schilbach, 2019). Storytelling allows people to share their emotional experiences, which is a typical manifestation of intentional emotional contagion in daily social interactions. The present study measured strangers’ behavioral and brain responses during the active processing of emotional story sharing. In conjunction with the Emotions as Social Information Model (van Kleef & Côté, 2022; van Kleef, 2009, 2016), this study also explored the mediating role of the degree of listeners’ intentional emotional contagion between emotion type and listeners’ behavioral performance.

#### Degree of conscious engagement on emotional contagion

Initial research on emotional contagion mainly focused on primary emotional contagion, in which individuals experienced similar emotional states by automatically and unconsciously mimicking others’ emotional expressions (Hatfield et al., 1994). However, as extant research indicates, a comprehensive understanding of emotional contagion necessitates the integration of automatic, unconscious sharing, and motivated processes. Specifically, individuals exhibit heightened sensitivity to the emotional states of those based on their social motivations (van Kleef & Côté, 2022).

Combined with Emotions as Social Information Model, the degree of conscious involvement of the listener may impact the effect of emotional contagion to some extent (Liu & Fu, 2022; van Kleef & Côté, 2022; van Kleef, 2009, 2016). The present study found that when listening to sad stories, the perspective-taking group had a significantly greater degree of emotional contagion than the direct-viewing group. This result indicated that the degree of intentional sadness contagion was stronger than the degree of primary sadness contagion, confirming that the degree of listener’s conscious involvement influenced on the degree of intentional sadness contagion. This indicates the existence of intentional sadness contagion.

#### Neural correlates of intentional sadness contagion: Dual attenuation of brain activation and interbrain synchrony

The results of single-brain activation in Experiment 2 showed that in the brain regions of the listener’s frontal pole, dorsolateral prefrontal cortex, angular gyrus, and supramarginal gyrus, the activation level when listening to sad stories was weaker than that of neutral stories. Simultaneously, the INS results indicated a weaker response when listening to sad stories compared to neutral stories on the channel pair of the speaker’s left frontal pole and listener’s right dlPFC, as well as the speaker’s left dlPFC and listener’s right dlPFC. We propose that it precisely captures a unique neural signature of intentional emotional contagion, distinct from primary emotional contagion. Intentional sadness contagion may not rely on instantaneous, mirroring-like emotional state matching, but rather initiates a top-down cognitive evaluation process.

As we know, during face-to-face conversations, individuals may think about others’ thoughts and mental states to predict their intentions and actions and then take appropriate actions to adapt to the environment (Suda et al., 2010). The mentalizing system, which involves cognitive control, emotion regulation, and attention allocation, plays a crucial role in helping individuals to understand others’ intentions and infer their mental activities based on each other’s gestures, behaviors, and facial expressions (Amodio & Frith, 2006; Grosse et al., 2020; Liang et al., 2022; Mayseless et al., 2019; Wang et al., 2018). From the perspective of cognitive resource allocation, listening to neutral stories may consume more cognitive resources, such as information processing and logical reasoning. Moreover, the reduced frontal activation may reflect individuals’ decreased self-referential immersion in sadness (Gusnard et al., 2001; Northoff et al., 2006), preventing emotional overload. Therefore, the mentalizing system was activated more during the processing of neutral stories. While the decreased INS may indicate strategic resource allocation (Neubauer & Fink, 2009) when the brain processes complex socioemotional tasks, although this result initially appears to contradict the traditional view that “INS reflects the strength of social connection” (Stephens et al., 2010). Consistent with the findings of Djalovski et al. (2021), close partners exhibited the highest behavioral synchronization but the lowest neural synchronization during empathy tasks, interpreted as an optimal resource allocation strategy that achieves efficient social performance at the cost of reduced neural alignment. In the present study, intentional sadness contagion between strangers may have triggered a similar adaptive neural reorganization. Rather than pursuing real-time neural alignment, the speaker and listener may redirect cognitive resources from emotional resonance to understanding others’ needs and constructing empathic concern.

In summary, this study demonstrates that reduced single brain activation and INS corresponding to intentional sadness contagion may mark an adaptive neurocognitive reorganization that facilitates prosocial behavior. This finding that may expand the functional interpretation of INS in social interaction.

#### Intentional sadness contagion increases interpersonal intimacy and facilitates prosocial behavior

The behavioral results of Experiment 1 indicated the presence of intentional emotional contagion. The behavioral results from Experiment 2 showed that the degree of self-other overlap induced by sad stories was greater than that of neutral stories. These results suggested that intentional sadness contagion may increase the listeners’ perceived interpersonal intimacy. When listeners had the viewpoint of perspective-taking (Hawk et al., 2011), they could consciously immerse their own emotional state in speakers. As a result, listeners’ self-focus decreased (Yogan, 2020) and shared representations were activated (Decety & Meyer, 2008) and then they felt closer to speakers. This result provides empirical evidence and implications from the perspective of intentional emotional contagion in the maintenance of interpersonal relationships.

Behavioral results from Experiment 2 also found that listeners exhibited more significant helping behaviors after speakers shared sad emotions than neutral emotions. This result demonstrated that intentional sadness contagion promoted prosocial behavior. A possible reason could be that listeners wanted to seek self-rewards associated with offering help, relieve their negative emotional states, and avoid being immersed in negative emotions (Batson et al., 2009, 2015). Similar research has also found that the facilitative effect of sad empathy is exclusively manifested among individuals with stronger perspective-taking abilities (Xu et al., 2019). People with higher emotional empathy are more willing to approach those presenting sad facial expressions and proactively extend potential assistance (Willis et al., 2015).

#### The mediating role of intentional sadness contagion in emotion type and self–other overlap

This experiment found a mediating role for the degree of intentional emotional contagion between emotion type and the degree of self-other overlap perceived by the listener. By increasing the degree of emotional contagion, sadness significantly enhanced the degree of self-other overlap between the speaker and listener. This finding illustrates the social interaction function of emotions and supports the theoretical perspective of the Emotions-as-Social-Information Model (van Kleef & Côté, 2022), which posits that a listener’s emotional responses mediate the effect of a speaker’s emotional expression on the listener’s behavior.

#### Limitations and future directions

This study also has several limitations that should be addressed in future research. First, its ecological validity could be enhanced, as we only employed a fixed, asymmetric “speaker-listener” interaction pattern. Future studies are encouraged to adopt a reciprocal, free “natural discussion” paradigm to explore interbrain neural synchrony of intentional emotional contagion in bidirectional natural communication. Second, the social interaction modalities were relatively simplistic. Incorporating natural cues (e.g., gaze direction, physical embrace) would better reflect real-world emotional exchange. Third, from an interdisciplinary perspective, future work could extend intentional emotional contagion research to political science, communication (e.g., how leaders’ emotional expressions shape followers’ attitudes; van Kleef & Côté, 2022), and artificial intelligence to inform human–machine interface development (van Kleef & Côté, 2022). This may fundamentally change the way humans interact with machines and enrich our understanding of how emotion regulates our daily life.

### Conclusions

This study investigates intentional emotional contagion based on story sharing. Our findings demonstrate a behavioral-neural dissociation in dyadic intentional sadness contagion: (a) intentional sadness contagion strengthens dyadic bonding by increasing interpersonal intimacy and promoting listeners’ prosocial responses, while (b) intentional sadness contagion engages distinct neural mechanisms, including reduced frontal and temporoparietal activity and weakened frontopolar-dlPFC INS. Mediation analysis fundamentally advances Emotions as Social Information model by identifying intentional emotional contagion mediated emotional communication and self-other overlap. Our findings suggest that intentional sadness contagion in dyadic social interaction has an adaptive neurocognitive reorganization which facilitates prosocial behavior.

## Supplementary Information

Below is the link to the electronic supplementary material.

Supplementary file1 (DOCX 697 KB)

## Data Availability

Data are available from the corresponding author on reasonable request.

## Code availability

All analyses were performed using MATLAB R2013b and R2022a with standard functions and toolboxes. All codes used are available upon reasonable request.
