# Supplementary material for: Intentional sadness contagion: Social bonding with neural decoupling
Source: Cogn Affect Behav Neurosci. 2026 May 19;26(4):1867–81. doi: 10.3758/s13415-026-01444-y (PMC13385017; doi:10.3758/s13415-026-01444-y)
Supplement: Supplementary file 1 — Supplementary file1 (DOCX 697 KB) [file 13415_2026_1444_MOESM1_ESM.docx]

**Pre-experiment: Screening Videos of Neutral and Sad Stories**

**1 Aim**

The experimental paradigm of this study was based on the “sender-receiver” communication mode (Wei et al., 2024). Therefore, we needed to prepare and screen the videos of stories in advance (Jospe et al., 2020).

**2 Procedure**

**2.1 Determination of the Story’s Themes**

In this study, stories with sad and neutral emotions that occured in individual’s own life were chosen as the carrier of intentional emotional contagion. Each speaker should tell a story for each theme. Specifically, the theme of the neutral story was “My Routine Day; Cleaning; Self-study”. For the sad stories, the themes were set as “Relatives’ illness or death; Alienation or breakdown of friendship; Failure in examinations”.

**2.2 Requirements for Storytelling**

Each speaker was required to recall a corresponding true story for each theme. Each theme was to be associated with a distinct short story. The narrative duration of each story should be two minutes (El Haj et al., 2021). The story themes must be familiar to college students (Gandolphe et al., 2018) and should be real-life events experienced by individuals. The description of each story should be detailed, including the occurring time and place, the emotional experiences of people involved and speaker (El Haj et al., 2021). Moreover, these events should happen recently (D’Argembeau & van der Linden, 2004).

And the speaker needed to engage as possible when she told the story. For example, facial expressions would play an important role in social interaction (Song et al., 2019). Speakers should be good at communicating their emotions through facial expressions, voice and intonation, and body language.

**2.3 Recruitment, Training and Recording of Speakers**

**Specific recruitment was as follows:**

In this study, the speakers were required to be outgoing who was good at expressing, not majoring in psychology, and able to complete the experiment carefully. In combination with these requirements, we have recruited a group of suitable speakers through classroom, poster, and Wechat moments. A total of 7 potential suitable speakers (22.43 ± 2.57 years, *M* ± *SD*) were recruited in the pre-experiment to tell the corresponding stories in their personal lives.

**The specific training process was as follows:**

The researcher sent the story theme and narration requirement (El Haj et al., 2021) to the 7 potential suitable speakers. Speakers should recall the corresponding story in her life according to the requirement. The speakers should write down each story. Speakers selected the interview time. When telling the story, speakers should be good at conveying their emotions through facial expressions, voice and intonation as well as body language. According to the speakers’ story, the researcher tried to provide some advice for the speakers to improve their story. After that, the speakers would send the story recording to the researcher. The researchers then listened to the recordings and gave feedback again.

**The video recording of the story is as follows:**

Finally, after the training, five potential suitable speakers (24.50 ± 1.95 years, *M* ± *SD*) recorded the storytelling, aiming to select two suitable speakers and use the video of their storytelling as the materials for the subsequent formal experiments.

During the recording phase, the speakers sat in front of the camera while brain activity of speakers was collected (Fig. s1). Neutral and sad stories were told separately in different block. In each block, the order of the stories was set by the speakers in order to help the speakers to reduce anxiety during storytelling and facilitate her engagement of the corresponding story.

The speakers needed to adjust her emotion to a neutral state before telling the story. After she adjusted her emotions, she could press the space bar and then heard the sound of “ding” as a reminder to start the storytelling. The countdown on the screen was used as a reminder of the time to tell the story, which would help them to improve the storytelling performance. The interval between two stories should be at least 30 seconds. The rhythm of storytelling was controlled by the speaker themselves. As the story was told, the brain activity of the speakers was recorded by LABNIRS (Shimadzu, Kyoto, Japan).

When the recording was complete, the researcher would use Adobe Premiere 2020 software to edit the recording videos. The specific operation would eliminate background noise and adjust each video to the appropriate size. All stories were edited into 2-minute video clips (El Haj et al., 2021). As a result, each speaker had three neutral stories and three sad stories.


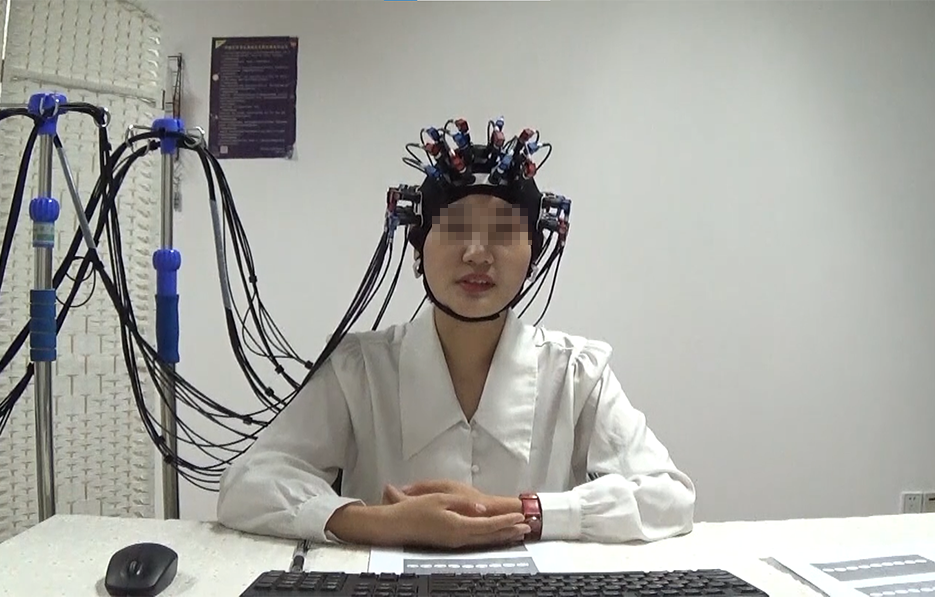


**Fig. s1 Speaker’s Brain Signal Acquisition**

**2.4 Rating of Storytelling Video Material**

A total of 17 females were recruited (one subject voluntarily withdrew from the experiment, and finally there were 16 valid females with age information of 20.44 ± 2.48 years (*M* ± *SD*)) to evaluate the video materials of all the stories told by the five speakers. All participants had normal or corrected-to-normal vision, and no history of brain injury or mental disorders. The participants signed an informed consent form before the experiment and received credit or compensation after the experiment. This study was approved by the Ethics Committee of the Department of Psychology, Renmin University, China.

The evaluation criteria consisted of five items: Q1: Evaluate the speaker’s physical attractiveness; Q2: Evaluate the speaker’s naturalness of storytelling; Q3: Evaluate the degree of emotional identification from the perspectives of both speaker and listener; Q4: Evaluate the speaker’s own emotional valence; and Q5: Evaluate the listener’s emotional valence induced by the story.

**3 Results**

Based on the above evaluation criteria, two speakers (24.00 ± 1.41 years, *M* ± *SD*) were ultimately selected as the final speakers. The stories of two emotions told by them matched well on each index. Bonferroni correction was used for post-hoc comparisons.

#### **3.1 Speaker’s Attractiveness**

A one-way repeated-measures ANOVA of the speakers’ attractiveness was performed. The results were as follows:

The main effect of speakers was significant (*F*(4, 60) = 4.23, *p* < 0.01, η^2^_p_ = 0.22). The pairwise comparison revealed that speaker 1 > speaker 2, *p* < 0.05; speaker 1 > speaker 5, *p* < 0.05; speaker 1 > speaker 3, *p* = 0.06; There was no significant difference in the attractiveness of speaker 2, speaker 3, speaker 4, and speaker 5 (*p* > 0.05).

There should be no significant difference in terms of speakers’ attractiveness. Hence, speaker 1 with higher attraction was deleted.

The descriptive statistics were presented in Table 1.

| **Table 1 Attraction of Five Speakers** | |
| --- | --- |
| **Speakers** | **Attraction (*M* ± *SD*)** |
|  |  |
| Speaker 1 | 7.06 ± 0.93 |
| Speaker 2 | 5.81 ± 1.17 |
| Speaker 3 | 6.06 ± 1.06 |
| Speaker 4 | 6.31 ± 1.30 |
| Speaker 5 | 5.75 ± 1.18 |

#### **3.2 Speaker’s Naturalness of Storytelling**

A one-way repeated-measures ANOVA of the natural degree of speakers’ storytelling was performed, and the results were as follows:

The main effect of speakers was significant (*F*(4, 60) = 6.30, *p* < 0.001, η^2^_p_ = 0.30). Naturalness of narration: speaker 2 > speaker 3, *p* < 0.01; speaker 4 > speaker 3, *p* < 0.05; The differences among speaker 1, speaker 2, speaker 4, and speaker 5 were not significant.

Therefore, according to the difference in the degree of naturalness, speaker 3 with a lower naturalness was deleted. Combined with attractiveness and naturalness, speaker 1 and speaker 3 were removed. Then, speaker 2, speaker 4, speaker 5 were the alternative choice.

The descriptive statistics were presented in Table 2.

| **Table 2 The naturalness of storytelling by the speakers** | |
| --- | --- |
| **Speakers** | **Naturalness (*M* ± *SD*)** |
|  |  |
| Speaker 1 | 7.06 ± 1.36 |
| Speaker 2 | 7.28 ± 0.90 |
| Speaker 3 | 6.27 ± 1.15 |
| Speaker 4 | 7.27 ± 1.03 |
| Speaker 5 | 6.82 ± 1.17 |

#### **3.3 I****dentification Degree of Emotion type**

A two-way repeated-measures ANOVA of speakers and emotion type was performed, and the results were as follows:

The main effect of speakers was significant (*F*(4, 60) = 2.54, *p* = 0.05, η^2^_p_ = 0.15). Pairwise comparison of the listeners’ identification about the speakers revealed that the difference was marginally significant between speaker 3 and speaker 2, *p* = 0.06. The difference between speaker 3 and 4 was also marginally significant, *p* = 0.07.

The main effect of emotion type was significant (*F*(1, 15) = 28.62, *p* < 0.001, η^2^_p_ = 0.66). Among them, the identification of neutral stories was significantly higher than that of sad stories (*p* < 0.001).

There was a significant interaction between speakers and emotion type (*F*(2.38, 35.76) = 5.14, *p* < 0.01, η^2^_p_ = 0.26). The results of simple effect were as follows:

When listening to neutral stories, speaker 2 and speaker 4 had almost the same identification degree.

When listening to sad stories, speaker 3 and speaker 2 had significant differences (*p* < 0.05), and speaker 3 and 4 also had significant differences (*p* < 0.05).

Therefore, according to the identification degree of emotion type, we cannot delete speakers other than speaker 1 and speaker 3. As a result, speaker 2, speaker 4, and speaker 5 as alternatives.

The descriptive statistics were presented in Table 3.

| **Table 3 Identification degree of emotion type** | | |
| --- | --- | --- |
| **Speakers** | **Identification rate (*M* ± *SD*)** | |
|  | **neutral** | **sad** |
| Speaker 1 | 0.96 ± 0.11 | 0.60 ± 0.37 |
| Speaker 2 | 0.98 ± 0.08 | 0.65 ± 0.28 |
| Speaker 3 | 1.00 ± 0.00 | 0.46 ± 0.38 |
| Speaker 4 | 0.98 ± 0.08 | 0.71 ± 0.27 |
| Speaker 5 | 0.96 ± 0.11 | 0.65 ± 0.31 |

#### **3.4 Speaker’s Own Emotion Valence**

A two-way repeated-measures ANOVA of speakers and emotion type was performed, and the results were as follows:

The main effect of speakers was significant (*F*(4, 60) = 7.30, *p* < 0.001, η^2^_p_ = 0.33). The pairwise comparison of speakers’ own emotion valence revealed that speaker 4 > speaker 2 (*p* < 0.05); The differences between speaker 2 and speaker 5(*p* > 0.05) as well as speaker 4 and speaker 5(*p* > 0.05) were not significant.

The main effect of emotion type was significant (*F*(1,15) = 368.01, *p* < 0.001, η^2^_p_ = 0.96). The valence score of speakers’ neutral story was greater than that of the speakers’ sad story.

The interaction was significant (*F*(4, 60) = 10.66, *p* < 0.001, η^2^_p_ = 0.42). The simple effect showed that:

When listening to neutral stories, there was no significant difference in the emotion valence of speaker 2 and speaker 4 (*p* > 0.05) as well as speaker 2 and speaker 5 (*p* > 0.05). There was also no significant difference in the emotion valence of speaker 4 and speaker 5 (*p* > 0.05).

When listening to sad stories, there was no significant difference in the emotion valence of speaker 2 and speaker 4 (*p* > 0.05) as well as speaker 2 and speaker 5 (*p* > 0.05). There was also no significant difference in the emotion valence of speaker 4 and speaker 5 (*p* > 0.05).

The descriptive statistics were presented in Table 4.

| **Table 4 emotion valence of speakers (*M* ± *SD*)** | | |
| --- | --- | --- |
| **Speakers** | **neutral** | **sad** |
| Speaker 1 | 5.27 ± 0.65 | 2.31 ± 0.71 |
| Speaker 2 | 5.31 ± 0.56 | 1.88 ± 0.70 |
| Speaker 3 | 5.10 ± 0.45 | 2.98 ± 0.79 |
| Speaker 4 | 5.58 ± 0.70 | 2.23 ± 0.83 |
| Speaker 5 | 5.25 ± 0.49 | 2.15 ± 0.64 |

According to the video recording, we also found that when speaker 4 told the two neutral story, she involuntarily smiled for more than 10 seconds. In order to eliminate the variation brought by speakers’ own unconscious expression, we finally selected speaker 2 and speaker 5.

#### **3.5** **Listener’s Emotion Valence Induced by the Stories**

A two-way repeated-measures ANOVA of speakers and emotion type was performed, and the results were as follows:

The main effect of speakers was significant (*F*(2.61, 39.13) = 6.86, *p* = 0.001, η^2^_p_ = 0.31). The emotion valence of listeners corresponding to speaker 4 was greater than that of speaker 2 (*p* < 0.05). The differences between speaker 2 and 5 as well as speaker 4 and 5 were also not significant (*p* > 0.05).

The main effect of emotion type was significant (*F*(1, 15) = 87.37, *p* < 0.001, η^2^_p_ = 0.85). The emotion valence of listening to neutral stories was higher than sad stories.

The interaction was significant (*F*(4, 60) = 6.75, *p* < 0.001, η^2^_p_ = 0.31). The results of simple effect showed that:

When listening to neutral stories, listener’s emotion valence induced by the stories of speaker 2 and 4 were significantly different (*p* < 0.05). There was no significant difference in listener’s emotion valence of speaker 2 and speaker 5 (*p* > 0.05) as well as speaker 4 and speaker 5 (*p* > 0.05).

When listening to sad stories, listener’s emotion valence induced by the stories of speaker 2 and 4 (*p* > 0.05). There was no significant difference in the emotion valence of listeners induced by speaker 2 and speaker 5 (*p* > 0.05) as well as speaker 4 and speaker 5 (*p* > 0.05).

The descriptive statistics were presented in Table 5.

| **Table 5 Listener’s Emotion Valence Induced by the Stories (*M* ± *SD*)** | | |
| --- | --- | --- |
| **Speakers** | **neutral** | **sad** |
| Speaker 1 | 5.29 ± 0.74 | 3.33 ± 0.89 |
| Speaker 2 | 4.88 ± 0.79 | 2.69 ± 0.85 |
| Speaker 3 | 4.92 ± 0.45 | 3.88 ± 0.84 |
| Speaker 4 | 5.40 ± 0.51 | 3.10 ± 0.81 |
| Speaker 5 | 5.04 ± 0.59 | 3.46 ± 0.98 |

In summary, the stories told by speaker 2 and speaker 5 (24.00 ± 1.41 years, *M* ± *SD*) were selected as the follow-up experimental materials.

**Reference**

D’Argembeau, A., & van der Linden, M. (2004). Phenomenal characteristics associated with projecting oneself back into the past and forward into the future: Influence of valence and temporal distance. *Consciousness and Cognition, 13*(4), 844–858. https://doi.org/10.1016/j.concog.2004.07.007

El Haj, M., Boutoleau-Bretonnière, C., & Janssen, S. M. J. (2021). Eye movements of recent and remote autobiographical memories: Fewer and longer lasting fixations during the retrieval of childhood memories. *Psychological Research, 85*(6), 2466–2473. https://doi.org/10.1007/s00426-020-01403-3

Gandolphe, M. C., Nandrino, J. L., Delelis, G., Ducro, C., Lavallee, A., Saloppe, X., Moustafa, A. A., & El Haj, M. (2018). Positive facial expressions during retrieval of self-defining memories. *Journal of Integrative Neuroscience, 17*(3), 281–286. https://doi.org/10.31083/JIN-170073

Jospe, K., Genzer, S., Klein Selle, N., Ong, D., Zaki, J., & Perry, A. (2020). The contribution of linguistic and visual cues to physiological synchrony and empathic accuracy. *Cortex, 132*, 296–308. https://doi.org/10.1016/j.cortex.2020.09.001

Song, J., Wei, Y. Q., & Ke, H. (2019). The effect of emotional information from eyes on empathy for pain: A subliminal ERP study. *PLoS One, 14*(12), e0226211. https://doi.org/10.1371/journal.pone.0226211

Wei, Y. Q., Zhang, L., Lian, T., Chen, Y., Liao, S. H., Guo, Q., & Hu, P. (2024). Happy storytelling promotes emotional contagion and interpersonal closeness. *Current Psychology, 43*(4), 2993–3001. https://doi.org/10.1007/s12144-023-04544-x
